# Venous Malformations in Childhood: Clinical, Histopathological and Genetics Update

**DOI:** 10.3390/dermatopathology8040050

**Published:** 2021-10-15

**Authors:** Isabel Colmenero, Nicole Knöpfel

**Affiliations:** 1 Department of Pathology, Hospital Infantil Universitario Niño Jesús, 28009 Madrid, Spain; 2 Department of Dermatology, Great Ormond Street Hospital for Children and UCL GOS Institute of Child Health, London WC1N 3JH, UK; nicole.knoepfel@crick.ac.uk; 3 Mosaicism and Precision Medicine Laboratory, The Francis Crick Institute, London WC1N 3JH, UK

**Keywords:** vascular anomalies, venous malformations, histopathology, genetics, somatic and germline mutations, targeted therapy

## Abstract

Our knowledge in vascular anomalies has grown tremendously in the past decade with the identification of key molecular pathways and genetic mutations that drive the development of vascular tumors and vascular malformations. This has led us to better understand the pathogenesis of vascular lesions, refine their diagnosis and update their classification while also exploring the opportunity for a targeted molecular treatment. This paper aims to provide an overview of venous malformations (VM) in childhood. Specific entities include common VMs, cutaneo-mucosal VM, blue rubber bleb nevus syndrome or Bean syndrome, glomuvenous malformation, cerebral cavernous malformation, familial intraosseous vascular malformation and verrucous venous malformation. The clinicopathological features and the molecular basis of each entity are reviewed.

## 1. Introduction

Venous malformations (VMs) are slow-flow vascular lesions that occur due to a defect in vascular morphogenesis during early embryonic life, sometime between 4 and 10 weeks of gestation. As a result, VMs are composed of ectatic venous channels with a thin or absent muscle wall. VMs are considered the most frequent vascular malformation referred to multidisciplinary centers that specialize in vascular anomalies, with an estimated incidence of 1 to 5 in 10,000 births [1,2]. They typically involve the skin, mucosa and subcutaneous tissue, but may also arise in deeper structures such as muscle, bone and internal organs. Most VMs are sporadic and isolated, but they can be part of complex vascular disorders such as blue rubber bleb nevus syndrome and the spectrum of overgrowth syndromes, among others. Somatic activating mutations in *TEK/TIE2* cause more than half of sporadic unifocal VMs [3,4], and somatic mutations in *PIK3CA* are responsible for around 20% of cases [5]. Mutations in both genes activate the PI3K/AKT/mTOR signalling pathway, underscoring the importance of this pathway in the pathogenesis and the opportunity of targeted molecular inhibitors for the treatment of VMs. However, TEK-mediated venous anomalies include a spectrum of phenotypes of varying severity and models of mutation acquisition that are distinctive to sporadic unifocal VM, inherited cutaneo-mucosal VM, sporadic multifocal VM and blue rubber bleb nevus syndrome [6]. 

In addition, the increasing advances in genetics uncovering the molecular mechanisms in vascular anomalies have contributed and without question will continue to play an essential role in the classification of vascular anomalies. One example is the identification of a somatic mutation in *MAP3K3* in the previously called “verrucous hemangioma”, which has been relocated to the VM group under the term “verrucous venous malformation” [7]. 

Most patients with VMs are diagnosed clinically, but when diagnosis is uncertain a biopsy for histopathology and genetic testing are recommended. 

VMs may still be found in the literature as “cavernous hemangioma” or “cavernoma”. This obsolete terminology should be avoided, as the suffix “oma” would suggest that VMs are neoplasms. Following the recommendations of the 2018 International Society for the Study of Vascular Anomalies (ISSVA) classification for vascular anomalies, we provide an outline of the sporadic and inherited conditions that encompass VMs (Table 1), discussing the clinicohistopathological features and molecular basis for each entity. 

## 2. Common VM 

Common VMs are mostly sporadic and unifocal, and account for more than 90% of VMs [2,8].

### 2.1. Clinical Features 

**Figure 1:**
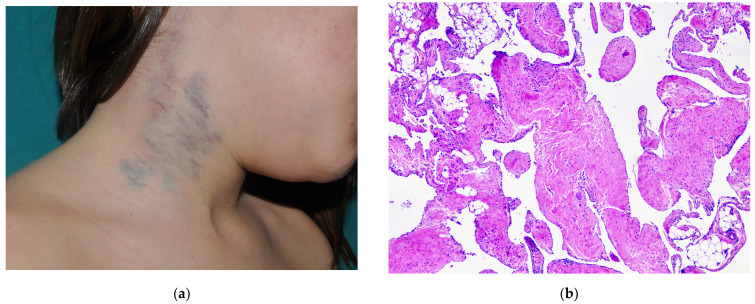

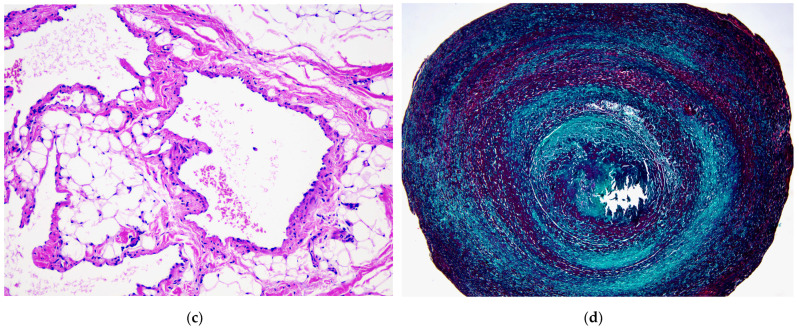
(**a**) Clinical appearance of a VM on the lateral neck. (**b**) A common VM showing large irregular vessels intersecting the tissue in a sponge-like fashion. Floating islands of normal tissue surrounded by endothelium are frequently seen. (**c**) One of the malformed venous channels showing a thin muscle wall and non-atypical endothelial cells lining the inner aspect of the vessel. (**d**) Pheboliths are characteristic of VMs.

VMs are usually noted at birth, but some cases become clinically evident later. They grow proportionally with the child and exhibit progressive ectasia with age. The most frequent location is the head and neck region (40%), followed by the extremities and trunk. The clinical presentation consists of a bluish-to-purple, soft and compressible nodule or mass, and its size may range from very small to extensive and deep lesions (Figure 1). Skin temperature is normal, and there is no thrill as these are slow-flow vascular malformations. As the dysmorphological features of VMs predispose to stagnant blood flow, these lesions can spontaneously thrombose and thus present with swelling and pain. On palpation, the presence of phleboliths (due to long-standing localized thrombosis) is pathognomonic for VM. A rapid expansion can be observed after trauma or hormonal modulation, typically during puberty or pregnancy when they tend to increase in size and become symptomatic. 

### 2.2. Genetics 

**Figure 2:**
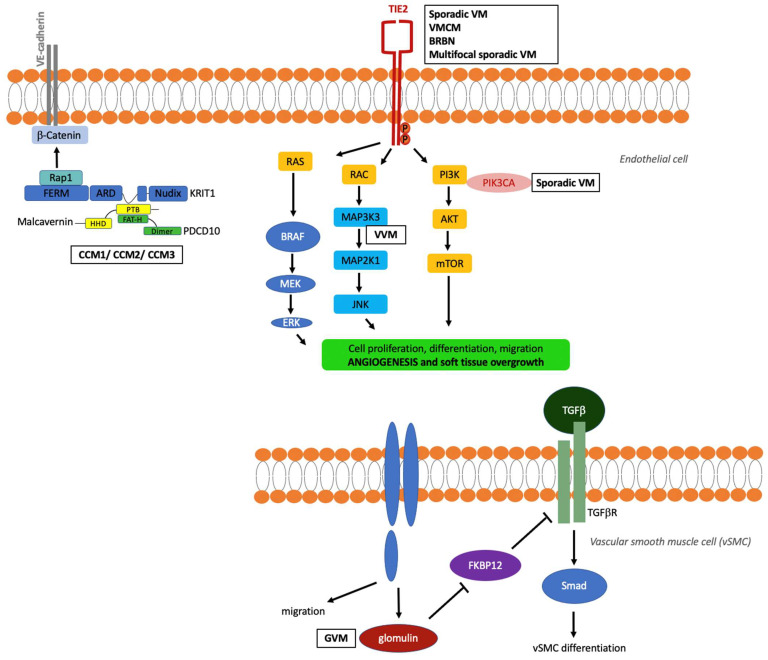
Diagram illustrating the genes and signalling pathways involved in venous malformations. VM, venous malformation; VMCM, cutaneo-mucosal venous malformation; BRBN, blue rubber bleb nevus; CCM, cerebral cavernous malformation; VVM, verrucous venous malformation; GVM, glomuvenous malformation. Adapted from ref. [12].

Somatic activating mutations in *TEK/TIE2*, the gene encoding endothelial cell tyrosine kinase receptor TIE2, cause 60% of sporadic unifocal VMs [3]. The most frequent mutation found in resected VM tissue is L914F (different from the inherited cutaneo-mucosal VM). Two major pathways involved in mediating the effects of TIE2 on endothelial cell function are the phosphoinositide 3-kinase (PI3K)/AKT and mitogen-activated protein kinase (MAPK) pathway (Figure 2) [9]. An estimated 20% of sporadic VMs are caused by somatic mutations in the *PIK3CA* gene [5]. In both cases, downstream signalling functions via the PI3K/AKT/mTOR pathway are responsible for regulating angiogenesis, proliferation, cell migration and vessel stability. The identification of genetic mutations and key molecular pathways that drive sporadic VMs have had a critical role in the understanding of the pathogenesis and use of targeted therapies in VMs (see prognosis and treatment) [10,11,12].

### 2.3. Histopathology 

Microscopically, VMs exhibit large, widely dilated venous channels of irregular shape and size, haphazardly arranged in the dermis, subcutis and deep soft tissues. These malformed vessels are lined by a single layer of flat endothelial cells with a concentrical muscle layer that is often focally absent or scant relative to luminal diameter (Figure 1). In some areas, the walls can show irregular strands or nodules of smooth muscle merging with fibromyxoid tissue, which is probably the consequence of organized thrombi. Lumens are empty or contain blood or organizing thrombi that may eventually become calcified (phleboliths). Papillary endothelial hyperplasia (Masson’s phenomenon) is a common finding. Vessels are separated by normal background tissue (Figure 2). Endothelial cells are diffusely positive for CD31, but CD34 staining is variable. Negative immunostaining for lymphatic markers such as D2-40 or PROX1 is helpful to differentiate VMs from lymphatic malformations, their main differential diagnosis. Similar to other malformations, WT1 is not expressed and the Ki-67 rate is extremely low [13,14,15].

### 2.4. Prognosis and Treatment

An estimated 40% of patients with a VM develop a localized intravascular coagulopathy characterized by elevated levels of D-dimers and normal-to-low levels of fibrinogen, which often correlates with the size and depth of lesions and the presence of phleboliths [1]. Depending on the size and location, VMs can be life-threatening because of bleeding, expansion or obstruction of vital structures; for example, when located in the oral mucosa and extending to the oropharynx and larynx, potentially compromising the airway [16]. However, the main complications associated with the slow expansion of VMs are aesthetically related, followed by chronic and significant pain. Regarding management, if patients are asymptomatic and there are no risks or associated complications, it is prudent to delay any therapeutic intervention and continue regular clinical follow-up. When treatment is contemplated, percutaneous intralesional sclerotherapy is the gold standard treatment of VM, alone or combined with surgical resection, to diminish the risk of recurrence. If the lesion is small and complete resection is possible without anatomic or functional consequences, surgical excision should be performed as the first choice. In complex VMs, the caring physicians are advised to take a multidisciplinary approach involving dermatologists, radiology interventionists, and surgeons involved in vascular anomalies, to determine the best treatment approach. However, these classical approaches have their limitations, including inaccessibility to challenging locations, and failure to completely eliminate the VM, therefore accounting for the persistence of the VM and regrowth over time. The identification of *TEK/TIE2* mutations in VMs has led to testing targeted therapy with the mTOR inhibitor rapamycin (sirolimus) [11,12]. Previous animal models of VM overexpressing mutant *TEK/TIE2* have shown that treatment with rapamycin reduced endothelial cell accumulation and development of VMs. Another model generated by injection of PIK3CA (H1047R)-expressing cells into mice led to the formation of highly vascularized and proliferative masses that reduced in size after everolimus treatment, though PI3K inhibitor, alpelisib (BYL719) resulted in a greater response. A prospective multicentric single-arm phase 3 trial (VASE) is currently ongoing to evaluate rapamycin efficacy in pediatric and adult patients with various slow-flow vascular malformations. The preliminary results for VMs suggest that rapamycin has a good effect in reduction of pain and/or in limitation (mobility or organ function), resulting in a general improvement rate of up to 89% [11]. 

## 3. Familial Cutaneo-Mucosal Venous Malformation (OMIM 600195)

The presence of multifocal VMs is often a clue to a familial form known as cutaneo-mucosal venous malformation (VMCM). This form accounts for 1–2% of VMs.

### 3.1. Clinical Features 

**Figure 3:**
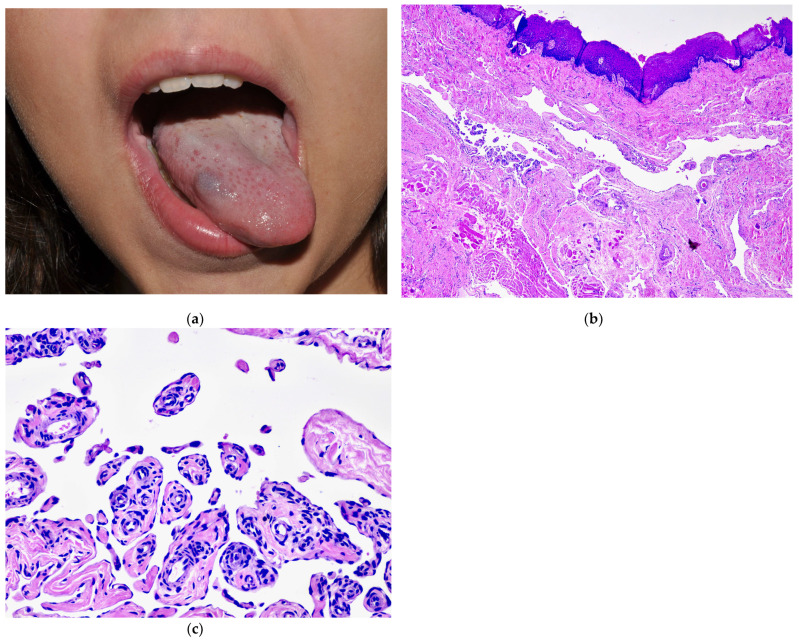
Clinical presentation of a VM in the setting of VMCM as a bluish soft nodule on the lateral tongue. This child also presented with multiple VMs on the skin. (**a**) Clinical presentation of a VM in the setting of VMCM as a bluish soft nodule on the lateral tongue. This child also presented with multiple VMs on the skin. (**b**) Irregular channels with absent muscle next to the mucosal surface. (**c**) Pseudopapillary projections within one of the lumens showing entrapped normal elements.

Patients with VMCM present a varying phenotype with multiple VMs on the skin and mucous membranes, most of them of small size, dome-shaped and with a bluish hue appearance (Figure 3). Family inspection is essential in this autosomal dominant disease, with an estimated penetrance of 90% by the age of 20 years [17]. 

### 3.2. Genetics

VMCM is caused by germline mutations in *TEK/TIE2* that also result in ligand-independent hyperphosphorylation of TIE2 and activation of the downstream PI3K/AKT pathway [18]. These activating mutations are most likely inherited as an autosomal-dominant familial trait. Several families have been found to carry a germline mutation causing a one amino acid substitution, R849W, but a second event (somatic) to the same gene is required to give rise to a VM [18,19]. 

More recently, a sporadic form of multifocal VM has been reported, which is also caused by *TEK/TIE2* mutations [6]. This newly defined entity termed multifocal sporadic VM (MSVM) appears to present as a milder phenotype of VMCM, without a family history of VMs. In this form, the mutation R915C is most frequently present as a mosaicism in blood, and a somatic Y897C mutation occurs at a different timepoint on the same gene allele [6].

### 3.3. Histopathology 

Features are similar to those of common VMs. Vessels are of small and medium size, and are lined by inconspicuous endothelial cells with flat or round nuclei. The smooth muscle coat is largely absent in most of the channels [20] (Figure 3).

### 3.4. Prognosis and Treatment 

The clinical management of VMs in VMCM does not differ from sporadic VMs, except for the importance on careful family history of vascular lesions consistent with autosomal dominant inheritance. Laboratory findings of D-dimer show elevated levels more often than in common VMs [9].

## 4. Blue Rubber Bleb Nevus Syndrome (OMIM 112200)

Blue rubber bleb nevus syndrome (BRBN) also known as Bean syndrome, is a rare sporadic disorder characterized by multiple cutaneous and internal VMs [21]. Patients present with a large number of lesions that increase in size and number with age, with a predilection for the skin, mucosae and gastrointestinal (GI) tract, though they can occur in any visceral organ.

### 4.1. Clinical Features 

**Figure 4:**
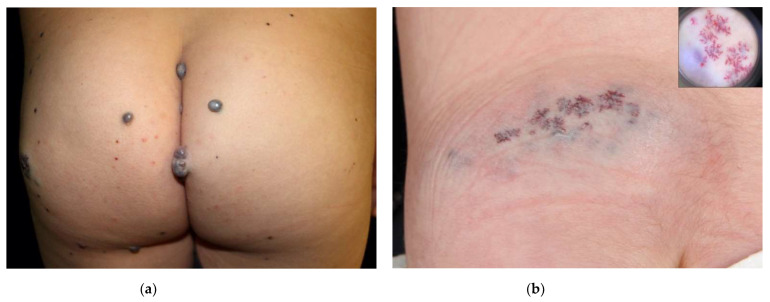

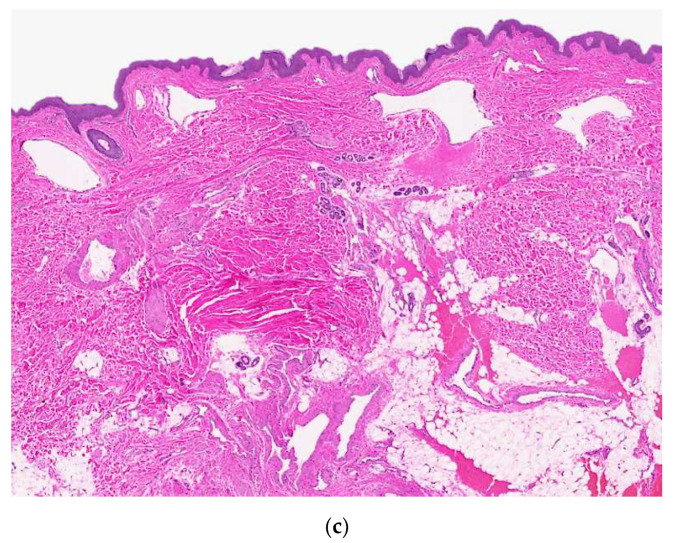
(**a**) Typical clinical presentation of BRBN with multiple small, soft, dome-shaped, bluish nodules. (**b**) Neonate with a large subcutaneous VM with a “fern-shaped” pattern. Dermoscopy (inset) highlights the central arborized pattern. Courtesy of Dr. Lisa Weibel. (**c**) Biopsy from the patient in (**b**) shows large abnormal channels with very thin muscle walls scattered all over the dermis. Note the larger and dilated vessels next to the epidermis, accounting for the fern-shaped appearance on the surface of the lesion. Genetic analysis of affected tissue identified doble (*cis*) mutations (T1105N-T1106P) confirming the diagnosis of BRBN. Courtesy of Dr. Peter Bode.

Cutaneous VMs in BRBN are characterized by small, dome-shaped, nipple-like bluish nodules with a rubbery consistency, hence the term “rubber bleb” (Figure 4). They occur on any surface of the skin and mucosae, and tend to aggregate and become hyperkeratotic on palms and soles. At some point, hundreds of lesions are found on the skin. Patients often exhibit a large VM, a so-called “dominant VM”, and in some cases a congenital single large VM with distinguishing features reported as central arborized-pattern or “fern-shaped” represents the first manifestation of BRBN (Figure 4) [22]. 

### 4.2. Genetics

In 2017, Soblet et al. identified two somatic double mutations (T1105N-T1106P) on the same allele *(cis)* in *TEK/TIE2* as the principal cause of BRBN [6]. In a cohort of 15 out of 17 patients with BRBN, deep sequencing reads from affected-tissue cDNA showed that all lesions from a given patient shared the same double mutations, hence in BRBN the cells are exclusively double mutant or wild-type, but never single mutant [6]. 

### 4.3. Histopathology 

Similar to other forms of VMs, the histological findings of the cutaneous lesions are large channels with thin walls having remarkably little or no smooth muscle. If a biopsy is taken from the “fern-shaped” areas of a large lesion, the dysmorphic channels can be seen very close to the epidermis (Figure 4). The deeper lesions have dysmorphic vessels with a discontinuous layer of smooth muscle, and the channels are separated by variable amounts of fibrous tissue. The intestinal lesions are mainly located at the submucosa with minimal involvement of the mucosa, and the muscular layer of the channels typically merges with the muscularis mucosa. The muscular layer of the bowel and the mesentery may be involved as well [23].

### 4.4. Prognosis and Treatment 

The prognosis of BRBN is dictated by the extent of intestinal involvement and the presence of other organ involvement. The GI lesions are typically located in the small intestine and exhibit a pathognomonic appearance under endoscopy. These can cause recurrent hemorrhage leading to chronic anemia, but patients may also develop other intestinal complications such as intussusception, volvulus and intestinal infarction [21]. The most common finding is symptomatic microcytic anemia due to chronic GI bleeding, requiring lifelong iron supplementation or repeated blood transfusions. Endoscopic treatment, as well as surgical excision, have proven to be beneficial to treat GI lesions; however, they are both ineffective in the long-term with high rate of lesion recurrence, especially in children [24]. 

In recent years, medical treatment with sirolimus (rapamycin) has shown an impressive improvement of GI bleeding with fast recovery of hemoglobin levels, and is currently considered the best therapeutic option when there is multi-organ involvement in BRBN [25,26]. The cutaneous lesions do not seem to respond to sirolimus in the same degree as the VMs in the GI tract [27]. Surgical removal of cutaneous lesions may be indicated due to cosmetic reasons or presence of symptoms such as pain. 

## 5. Glomuvenous Malformation (OMIM 138000)

Glomuvenous malformation (GVM), previously considered a variant of glomus tumour, is best regarded as a peculiar type of venous malformation with glomus cells in the wall of the malformed veins. 

GVMs represent 5% of the venous anomalies and are familial in more than 60% of the cases [17]. 

### 5.1. Clinical Features

**Figure 5:**
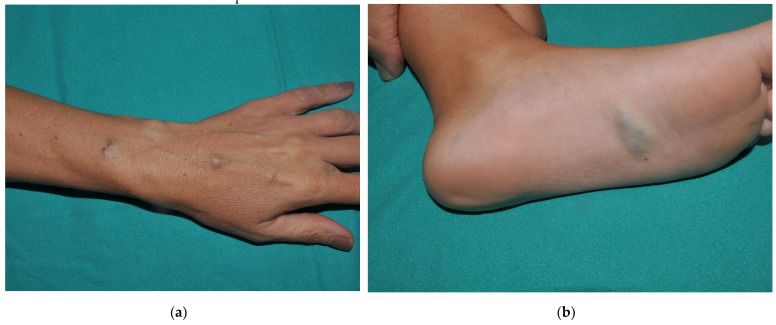

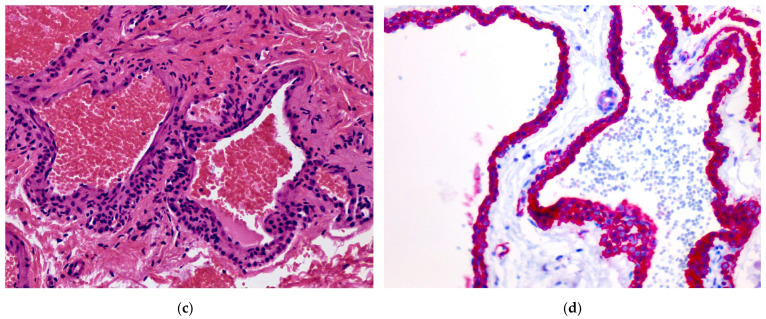
(**a**) Clinical presentation of GVM as multiple bluish-to-purplish papulo-nodular lesions in a mother and (**b**) daughter, as plaque-type GVM. (**c**) A group of dilated vascular channels showing monomorphic glomus cells in the wall. (**d**) Glomus cells are strongly positive for SMA.

GVMs present as multiple purplish-blue macular to papular and nodular lesions that can appear isolated or grouped following a segmental distribution known as plaque-type GVM (Figure 5). They are characterized by being tender on palpation and are not compressible. Paroxysms of pain can occur either spontaneously or evoked by trauma or compression. The congenital extensive GVM presents with a peculiar phenotype in newborns being more pinkish in colour and showing an atrophic appearance that can be misdiagnosed as a capillary malformation. Over time, lesions become more bluish in colour. The number of lesions varies among family members, some having only few lesions while others present hundreds of them. 

### 5.2. Genetics

GVMs can be either sporadic or most commonly autosomal dominant inherited. GVMs are caused by several loss-of-function mutations in the *glomulin* gene, located on chromosome 1p21-22 (OMIM 601749) [28]. The expressivity is variable, and penetrance is incomplete. Somatic second hits explain the wide phenotypic variability of GVMs in patients with inherited GVMs. The most common second hit appears to be an acquired uniparental isodisomy of chromosome 1p [28,29,30]. Superimposed mosaic (linear or plaque-type) lesions can appear in this setting [31]. Mutations in *glomulin* are not a feature of classic glomus tumors. 

### 5.3. Histopathology

GVMs show venous-like dysplastic channels of variable size lined by flattened endothelium and surrounded by one or more layers of glomus cells. The glomus cell component may vary widely between regions, and some microscopic fields may show only veins devoid of glomus cells. GVMs with smooth muscle cells have been designated as glomangiomyomas [32]. Due to their derivation from vascular smooth-muscle cells, glomus cells in GVMs are positive for smooth muscle α-actin (SMA), muscle specific actin (MSA), h-caldesmon and vimentin (Figure 5).

### 5.4. Prognosis and Treatment

New lesions may appear over time. Elastic compressive garments often aggravate the pain. Surgery can be considered for cosmetically disturbing solitary lesions. Nd:YAG laser therapy and sclerotherapy have shown to be a successful treatment in some cases [33,34,35,36].

## 6. Cerebral-Cavernous Malformation (OMIM 116860)

Cerebral-cavernous malformation (CCM) is a vascular disorder that affects up to 0.5% of the total population [37]. The vascular lesions encountered in CCM are frequently referred to as “cerebral cavernomas”, and they arise primarily in the central nervous system (CNS), though they can affect at a lower frequency the retina, liver, kidney, and skin [38,39,40].

### 6.1. Clinical Features

Many affected individuals are clinically asymptomatic during their entire lives, but patients present an increased risk for stroke, seizures, motor and sensory deficits, and headaches [40,41,42]. CCM usually manifests between 20 to 30 years of age, but clinical manifestations can occur at any age.

**Figure 6:**
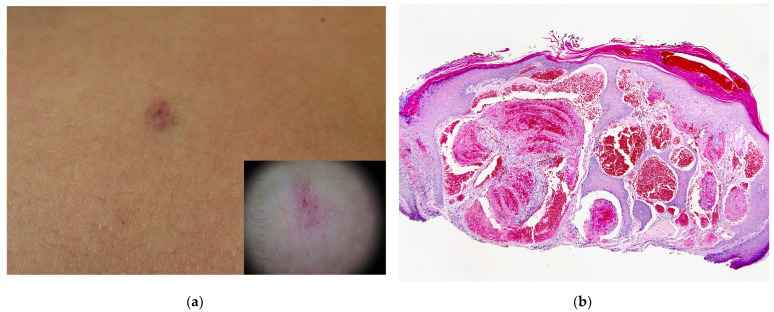
(**a**) Clinical presentation of a cutaneous vascular lesion in the setting of CCM known as capillary malformation of punctate type. Dermoscopy (inset) highlights a dotted vascular pattern. Courtesy of Dr. Ana Martin-Santiago. (**b**) Dilated thin-walled vessels expand the papillary dermis in this superficial biopsy of a hyperkeratotic lesion in a patient with CCM. The epidermis shows acanthosis and elongation of the rete ridges, together with hyperkeratosis. Thrombi are frequently seen within the lumens. Courtesy of Carles Saus.

Cutaneous vascular malformations are estimated to be present in around 9% of CCM patients. Three distinct major cutaneous vascular malformations phenotypes have been described: hyperkeratotic cutaneous capillary venous malformations (39%), capillary malformations (34%) (Figure 6) and venous malformations (21%) [43].

Hyperkeratotic cutaneous capillary venous malformations are congenital and mostly located on the limbs. They are plaque-like, more or less thick, irregularly shaped, and black or crimson coloured with bluish discolouration of the peripheral skin [43].

Capillary malformations are usually congenital and appear as a port wine stain or so called “punctate” capillary malformation [43].

Venous malformations in patients with CCM may appear as single or multiple nodules. Single lesions are frequently located on a limb, and multiple lesions affect the head and neck, trunk and limbs. Most lesions are not present at birth, and new lesions may emerge into adulthood. Depending on the depth of cutaneous involvement, some large subcutaneous nodules are colourless. Lesions range from a few millimeters (superficial lesions) to 5 cm (subcutaneous nodules). 

### 6.2. Genetics

About 20% of CCMs are inherited because of familial mutations in CCM genes while 80% of CCMs occur without a positive family history. CCM is transmitted as an autosomal dominant trait with incomplete penetrance. Mutations in *CCM1/KRIT1* (krev interaction trapped 1, OMIM: 604214), *CCM2/MGC4607* (encoding a protein named malcavernin, OMIM: 607929), and *CCM3/PDCD10* (programmed cell death 10, OMIM: 609118) cause cerebral cavernous malformations type 1 (OMIM: 116860), type 2 (OMIM: 603284), and type 3 (OMIM: 603285), respectively. All the mutations identified in these genes cause a loss of function and compromise the protein functions needed for maintaining the vascular barrier integrity. Loss of function of CCM proteins causes molecular disorganization and dysfunction of endothelial adherens junctions [44]. CCM1 is the most frequently mutated gene in CCM patients with cutaneous vascular malformations [43]. Recently, somatic mutations of *MAP3K3*, *PIK3CA*, *MAP2K7*, and CCM genes have been identified in cerebral CCM lesions, suggesting that CCM may also present as a mosaic disorder [37].

### 6.3. Histopathology

CNS lesions are usually small and composed of compact masses of large thin-walled blood vessels with collagenous walls lacking smooth muscle, often in a back-to-back arrangement. Organizing thrombi are common. Lesions are surrounded by a rim of brain parenchyma with gliosis and hemosiderin deposition [45]. 

Histological examination of hyperkeratotic cutaneous capillary venous malformations shows one superficial component with acanthosis and hyperkeratosis of the epidermis, associated with dilated vessels in the upper dermis. A second deeper component is characterized by dilated capillaries and veins in the deep dermis and hypodermis (Figure 6). The walls of the venous vessels contain two to several layers of smooth-muscle cells, readily visible on haematoxylin–eosin stained sections [43].

The venous malformations in patients with CCM are well-circumscribed lesions in the dermis, made of closely packed, enlarged, thin-walled vessels, without intervening normal cutaneous components. This nodular histological pattern is the same in CNS lesions [43].

### 6.4. Prognosis and Treatment

A comparison between the 3 CCM genes has revealed that CCM3 patients have an increased risk of hemorrhage, particularly during childhood. In fact, germline mutations in *PDCD10* predispose patients to a more severe form of CCM disease [41,46]. Treatment strategies fall into 2 categories: surgical removal and symptom relief. Lesions causing disabling seizures and/or focal neurologic deficits and/or cerebral hemorrhages need to be removed whenever possible. Medical treatment is recommended in case of seizures and headaches. Acetylsalicylic acid, heparin and warfarin may increase the risk of hemorrhage. Regarding the management of cutaneous vascular lesions in the setting of CCM, surgical removal or laser treatment may be indicated in case of cosmetic concerns. 

## 7. Familial Intraosseous Vascular Malformation (OMIM 606893)

Familial intraosseous vascular malformation (VMOS) is a biologically aggressive intraosseous form of VM associated with ELMO-2 gene mutations. Abnormally enlarged blood vessels specifically involve membranous bone, resulting in dysregulated bone remodeling [47,48].

### 7.1. Clinical Features

Patients with VMOS present with life-threatening progressive expansion of the mandible, maxilla or other craniofacial bones. Clavicle, ribs, and vertebrae can also be affected. In some cases, bone lesions are accompanied by midline abnormalities such as diastasis recti and supraumbilical raphe. 

Prior to the onset of puberty, the lesion is restricted to the mandibular and maxillary region; thereafter, rapid expansion occurs, and lesions extend to all cranial bones, causing an increase in intracranial pressure or massive bleeding that can be life threatening.

### 7.2. Genetics

VMOS is an autosomal-recessive condition caused by loss of function homozygous mutations in the Engulfment and cell motility protein 2 (*ELMO2*) gene located in chromosome 20. Absence of *ELMO2* correlates with a significant downregulation of binding partner DOCK1, resulting in deficient RAC1-dependent cell migration [47].

### 7.3. Histopathology

As no pathognomonic radiographic findings have been reported, pathological findings are critical for diagnosis of VMOS. The abnormally dilated blood vessels that expand and destroy the bone are lined by bland endothelial cells (CD31+, Ki67-) and surrounded by a thin smooth muscle layer demonstrating an immature phenotype. Abundant mature fatty tissue is present between the abnormal venous channels. On immunohistochemistry, the muscle cells are positive for smooth muscle actin (SMA); however, desmin and h-caldesmon—considered markers for mature vascular smooth muscle cells—are negative. Given that h-caldesmon tethers actin and myosin in smooth muscle cells for regulation of muscle tone [49], it has been hypothesised that the h-caldesmon negative immature smooth muscle cells in VMOS are incapable of withstanding blood pressure, causing dilatation [50].

### 7.4. Prognosis and Treatment

VMOS is a type of intraosseous vascular malformation with aggressive biological and challenging management. As the disease rapidly progresses as the affected individual grows, surgical interventions should be taken into consideration before the initiation of complications. Close follow-up is necessary for determining intracranial and orbital involvement to prevent complications such as exophthalmos, dystopia, and vision loss. Total or near-total surgical resection should be taken into consideration, following endovascular embolization, before orbital and cranial base involvement takes place.

Early placement of a ventriculoperitoneal shunt to prevent intracranial pressure elevation may be lifesaving. Full-mouth tooth extraction should be taken into consideration to prevent life-threatening recurrent gingival episodes. Percutaneous administration of 99% ethyl alcohol may be used to prevent postoperative bleeding in the surgical site [50].

## 8. Verrucous Venous Malformation (Formerly Verrucous Hemangioma)

The term verrucous venous malformation (VVM) refers to vascular lesions consisting of a dermal and subcutaneous vascular component associated with an overlying verrucous surface. The nature of VVM has been controversial. Based on the clinical features, Imperial and Helwig [51] initially considered VVM to be a vascular malformation involving the subcutaneous tissue associated with reactive epidermal acanthosis and hyperkeratosis. Based on histopathological findings such as thick-walled vessels, multilamellated basement membrane, positive staining for Wilms tumour 1 protein (WT1) and glucose transporter 1 protein (GLUT1), some authors have found it difficult to exclude a neoplastic nature [52,53,54,55,56]. The recent identification of a genetic mutation in *MAP3K3*, downstream of the ANG1-TIE2 pathway supports the classification of this lesion as a VM [7]. 

### 8.1. Clinical Features 

**Figure 7:**
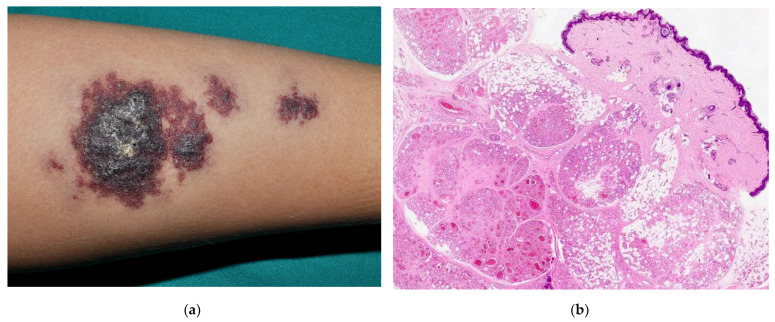

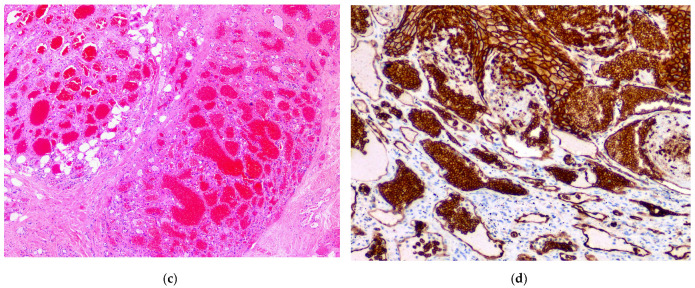
(**a**) Clinical presentation of VVM as grouped hyperkeratotic violaceous plaques on the lower limb. (**b**) Low power view of a VVM showing a superficial vascular component associated with verrucous hyperplasia and hyperkeratosis. The deep portion of the lesions is composed of vessels organized in lobules. (**c**) The lobules show variably dilated vessels and fibrosis. (**d**) GLUT1 positivity is a feature of VVM.

VVMs are present at birth or appear early during infancy. The most common locations are the limbs, especially lower limbs [52]. As most vascular malformations, they exhibit proportional growth with the child. The typical clinical presentation of VVM consists of well-circumscribed purple and hyperkeratotic linear plaques ranging in size from 2.5 to 20 cm in diameter (Figure 7). In young patients, lesions are non-keratotic, soft, and bluish-red, but they become increasingly hyperkeratotic over time. A subcutaneous variant of VVM has been recently reported, presenting as deep-seated bluish nodules [57]. 

### 8.2. Genetics 

VVM is a non-hereditary venous malformation caused by mosaic missense mutations in mitogen-activated protein kinase kinase kinase (*MAP3K3*), which is involved in the angiopoietin 1 (ANG1) and tunica internal endothelial cell kinase (TIE2) signalling pathway [7]. 

### 8.3. Histopathology 

A typical case of VVM shows compact hyperkeratosis, papillomatosis and irregular acanthosis overlying dilated vessels that involve the superficial dermis, extending to the deep dermis and subcutaneous tissue. In the deep part of the lesion, the vessels are organized in lobules (Figure 7). Thick-walled round capillaries/venules with a multilayered basement membrane, closely resembling those seen in IH in its involutive phase, are frequently observed. Endothelial cells of VVM are reactive for panendothelial markers and WT1, and show cytoplasmic immunoreactivity for GLUT1, which is usually focal in contrast to the diffuse staining seeing in IH. The lymphatic endothelial marker D2-40 can be focally positive.

VVM can be clinically confused with “angiokeratoma”, a vascular anomaly of uncertain nature, that lacks the deep vascular components present in VVM. Lymphatic malformations can also show varying degrees of hyperkeratosis and acanthosis, but they lack the lobular arrangement of the vessels seen in most VVMs and are negative for GLUT1 and WT1 [58].

### 8.4. Prognosis and Treatment

Treatment of VVM is mainly surgical, in a single procedure or in stages. VVM requires wide excision because it usually extends deep into the subcutis and slightly beyond its verrucous surface. Treatment can be quite challenging in lesions involving an extensive anatomic area as recurrence is very common when surgical resection is incomplete. Laser treatment has also been used with good results [59].

## 9. Others 

VMs can be part of complex vascular disorders, such as the Klippel–Trenaunay (OMIM 149000), Servelle–Martorell, Maffucci (OMIM 614569), CLOVES (OMIM 612918), Proteus (OMIM 176920) and Bannayan–Riley–Ruvalcaba (OMIM 158350) syndrome. Table 2 summarizes the associated anomalies and genetics for each syndrome. 

## 10. Conclusions

Venous malformations represent a heterogenous group of lesions presenting in the skin, soft tissues and sometimes in viscerae. Some histopathological features and the clinicopathological correlation are essential to properly classify the lesions, as they have distinctive features, genetic background, prognosis and treatment. Genetic testing is a helpful tool in the diagnosis of challenging cases where clinicopathological features are not typical.

## Figures and Tables

**Table 1:** ISSVA classification for vascular anomalies—venous malformations, last revision May 2018.

Venous Malformations (VMs)	Genetics
Common VM	*TEK* (TIE2)/*PIK3CA*
Familial VM cutaneo-mucosal (VMCM)	*TEK* (TIE2)
Blue rubber bleb nevus (Bean) syndrome	*TEK* (TIE2)
Glomuvenous malformation (GVM)	*Glomulin*
Cerebral cavernous malformation (CCM)	CCM1—*KRIT1*, CCM2—*Malcavernin*, CCM3—*PDCD10*
Familial intraosseous vascular malformation (VMOS)	*ELMO2*
Verrucous venous malformation (formerly verrucous hemangioma)	*MAP3K3*
Others	

Available at https://www.issva.org/classification (accessed on 28 March 2021).

**Table 2:** Other syndromes associated with venous malformations.

	Associated Anomalies	Genetics
Klippel–Trenaunay syndrome	CM + VM +/− LM + limb overgrowth	*PIK3CA*
Servelle–Martorell syndrome	Limb VM + bone undergrowth	
Maffucci syndrome	VM +/− spindle-cell hemangioma + enchondroma	*IDH1 /IDH2*
CLOVES syndrome	LM + VM + CM +/− AVM + lipomatous overgrowth	*PIK3CA*
Proteus syndrome	CM, VM and/or LM + asymmetric somatic overgrowth	*AKT1*
Bannayan–Riley–Ruvalcaba syndrome	AVM + VM + macrocephaly, lipomatous overgrowth	*PTEN*

CM, capillary malformation; LM, lymphatic malformation; AVM; arteriovenous malformation; CLOVES, Congenital, Lipomatous, Overgrowth, Vascular malformations, Epidermal nevi and Spinal/skeletal anomalies and/or Scoliosis.
